# Toxicity of ten herbicides to the tropical marine microalgae *Rhodomonas salina*

**DOI:** 10.1038/s41598-020-64116-y

**Published:** 2020-05-06

**Authors:** Marie C. Thomas, Florita Flores, Sarit Kaserzon, Rebecca Fisher, Andrew P. Negri

**Affiliations:** 1 Australian Institute of Marine Science, Townsville, QLD 4810 Australia; 2 AIMS@JCU: Australian Institute of Marine Science, College of Marine and Environmental Sciences, James Cook University, Townsville, Queensland 4811 Australia; 3 Queensland Alliance for Environmental Health Sciences (QAEHS), The University of Queensland, Woolloongabba, QLD 4102 Australia; 4 Australian Institute of Marine Science, Indian Ocean Marine Research Centre, University of Western Australia, Crawley, WA 6009 Australia

**Keywords:** Environmental impact, Marine biology, Microbial ecology

## Abstract

Herbicide contamination of nearshore tropical marine ecosystems is widespread and persistent; however, risks posed by most ‘alternative’ herbicides to tropical marine microalgae remain poorly understood. Experimental exposures of the important but understudied microalgae *Rhodomonas salina* to seven individual Photosystem II (PSII) inhibitor herbicides (diuron, metribuzin, hexazinone, tebuthiuron, bromacil, simazine, propazine) led to inhibition of effective quantum yield (ΔF/F_m_′) and subsequent reductions in specific growth rates (SGR). The concentrations which reduced ΔF/F_m_′ by 50% (EC_50_) ranged from 1.71-59.2 µg L^−1^, while the EC_50_s for SGR were 4-times higher, ranging from 6.27-188 µg L^−1^. Inhibition of ΔF/F_m_′ indicated reduced photosynthetic capacity, and this correlated linearly with reduced SGR (R^2^ = 0.89), supporting the application of ∆F/F_m_’ inhibition as a robust and sensitive indicator of sub-lethal toxicity of PSII inhibitors for this microalga. The three non-PSII inhibitor herbicides (imazapic, haloxyfop and 2,4-Dichlorophenoxyacetic acid (2,4-D)) caused low or no toxic responses to the function of the PSII or growth at the highest concentrations tested suggesting these herbicides pose little risk to *R. salina*. This study highlights the suitability of including *R. salina* in future species sensitivity distributions (SSDs) to support water quality guideline development for the management of herbicide contamination in tropical marine ecosystems.

## Introduction

## Herbicides in tropical marine ecosystems

Poor water quality, including pesticide contamination, has long been recognized as a threat to the health and resilience of tropical and subtropical marine ecosystems (Asia Pacific^1–5^, Central America^6^, Mexico^7^, Caribbean^8^). In north Queensland, Australia, herbicide transport from agriculture runoff into coastal waters peaks with summer rainfall^9^; however, the persistence of many herbicides^10^ contributes to herbicide detection year-round in the Great Barrier Reef (GBR) catchment area^11–13^. The GBR represents the most studied tropical marine location for herbicide contamination^14^, with annual monitoring conducted by the Marine Monitoring Program (MMP) under the Reef 2050 Long-Term Sustainability Plan assessing long-term trends in water quality in coastal waters of Queensland and the GBR marine park^13,15^.

Five Photosystem II (PSII) inhibitor herbicides ametryn, atrazine, diuron, hexazinone, and tebuthiuron are the most frequently detected herbicides in GBR waters. For example, maximum sustained concentrations of 778 ng L^−1^ diuron, 405 ng L^−1^ atrazine and 134 ng L^−1^ hexazinone have been reported in recent monitoring programs using passive sampling techniques. However, peak concentrations of up to 22 µg L^−1^ diuron have previously been identified in grab samples during flood events^16^. These five herbicides have therefore been identified as ‘priority’ herbicides for reductions by management^12–14,17,18^. PSII inhibitor herbicides specifically target the PSII of the photosynthetic complex within chloroplasts by competing with plastoquinone for the Q_B_ binding site on D1 proteins within the PSII^19^. This leads to the blocking of the light-induced electron transport chain, reducing photosynthetic efficiency and causing oxidative damage to PSII^19^. Since PSII is common across photosynthetic organisms, these herbicides can impact non-target marine species, including corals^20,21^, microalgae^22,23^, crustose coralline algae^24^, foraminifera^25^, and seagrass^26–29^. Reduced photosynthetic efficiency and damage to PSII caused by PSII inhibitor herbicides results in ecologically-relevant effects, such as inhibition of growth in microalgae^23^ and seagrass^30^, and bleaching, partial colony mortality, and reduced fecundity in coral^31^.

## Potential risks of ‘alternative’ herbicides

The risks posed by priority PSII inhibitor herbicides in runoff have led Australian regulators to tighten registrations and regulations for the application of some of these herbicides in agriculture^32^. As a result, there is a transition towards the application of ‘alternative’ PSII and non-PSII inhibitor herbicides in coastal agriculture^33^. Compared to PSII inhibitor herbicides, non-PSII inhibitor herbicides exhibit a range of different modes of action, such as inhibiting acetohydroxyacid synthase (AHAS)^34,35^ or acetyl-CoA carboxylase (ACCase)^36^ that result in reduced cell growth in plants. In recent years, 55 pesticides, including the five priority PSII inhibitor herbicides, as well as eight alternative PSII inhibitor herbicides (bromacil, fluometuron, metribuzin, terbuthylazine, propazine, simazine, terbutryn, prometryn) and eight alternative non-PSII inhibitor herbicides (2,4-D, fluroxypyr, monochlorophenoxyacetic acid (MCPA), imazapic, metsulfuron-methyl, metolachlor, haloxyfop, fluazifop) (Table 1) have been detected in the GBR and its catchments^17,37^. While most alternative herbicides detected in coastal waters of the GBR are registered for use in agriculture, these herbicides have only recently been added to pesticide analytical suites conducted as part of GBR MMP^38^. Many of the alternative herbicides occur at frequencies and concentrations similar to the regulated priority PSII inhibitor herbicides they are replacing (but usually <1 µg L^−1^)^13,17,37^ and often exhibit similar chemical properties and toxicities (Table S1). However, knowledge of the likely ecological effects of alternative herbicides in GBR waters is limited and toxicity data that underpins their registrations are generally scarce^39^.

**Table 1:** Summary of detected herbicides in the GBR and its catchment. Comparison of water quality guideline values (WQGVs)^39^ (all of low reliability) based on freshwater species and proposed water quality guideline values (PGVs)^43,44,93^ for 99%, 95%, 90% and 80% species protection (based on marine and freshwater species) against toxicity thresholds [no effect concentration (NEC); effect concentration inhibiting the specific growth rate by 10% (EC_10)_)] values derived for  *Rhodomonas salina* in this study (from Table 3). All concentrations in µg L^−1^. NA signifies no available guideline values. Bold indicates herbicides tested in this study.

Herbicide	Mode of action	WQGVs				PGVs				Guideline reliability	NEC This study	EC_10_ This study
		PC99	PC95	PC90	PC80	PC99	PC95	PC90	PC80			
*‘Priority’ PSII inhibitor herbicides*												
Diuron	Inhibition of photosynthesis at PSII	0.2*				**0.43**	**0.67**	**0.86**	**1.2**	**Very high**	**1.7**	**1.9**
Hexazinone		75*				**1.8**	**2.5**	**3.1**	**4**	**Low**	**4.6**	**4.0**
Tebuthiuron		**0.02**	**2.2**	**20**	**160**	**4.7**	**11**	**17**	**26**	**Moderate**	**23**	**28**
Ametryn		0.5	1	1.6		0.10	0.61	1.3	2.8	Low		
Atrazine		0.7	13	45	150	NA				NA		
*‘Alternative’ PSII inhibitor herbicides*												
Bromacil	Inhibition of photosynthesis at PSII	180*				**0.23**	**1.1**	**2.2**	**4.8**	**Moderate**	**5.5**	**4.9**
Fluometuron		NA				20	40	55	77	Low		
Metribuzin		**NA**				**2**	**2.7**	**3.1**	**3.9**	**Moderate**	**2.2**	**2.7**
Terbuthylazine		NA				0.40	0.97	1.6	2.8	Moderate		
Propazine		**NA**				**2.2**	**4.6**	**6.4**	**9.2**	**Low**	**28**	**42**
Simazine		**0.2**	**3.2**	**11**	**35**	**28**	**63**	**84**	**130**	**Low**	**48**	**38**
Terbutryn		NA				0.079	0.26	0.51	1.2	Moderate		
Prometryn		NA				0.11	0.52	1.1	2.2	Low		
*‘Alternative’ non-PSII inhibitor herbicides*												
2,4-D	Auxin mimic, promotes uncontrolled growth	**140**	**280**	**450**	**830**	**1,000**	**2,500**	**3,800**	**5,800**	**Low**	**>279,000**	**>279,000**
Fluroxypyr		NA				87	200	290	440	Low		
MCPA		1.4*				1	17	60	240	Low		
Imazapic	Inhibition of AHAS	NA				**0.049**	**0.44**	**1.2**	**3.6**	**Very low**	**363,000**	**410,000**
Metsulfuron-methyl		NA				NA				NA		
Metolachlor	Inhibition of cell division	NA				NA				NA		
Haloxyfop	Inhibition of ACCase	**NA**	**590**	**2,000**	**3,400**	**590**	**2,000**	**3,400**	**6,100**	**Low**	**>3,700**	**>3,700**
Fluazifop		NA				NA				NA		

*Level of protection unknown.

Water quality guideline values (WQGVs) exist for only a handful of alternative herbicides in Australia^39^ or globally (e.g. Canada^40^, EU^41^). According to the Australian and New Zealand Guidelines for Fresh and Marine Water Quality (ANZG)^39^, there are currently only four WQGVs for alternative PSII and non-PSII inhibitor herbicides (bromacil, simazine, 2,4-D, MCPA) and five guidelines for the priority herbicides (diuron, atrazine, ametryn, tebuthiuron, hexazinone) detected in GBR waters; however, all were derived from freshwater toxicity thresholds and are of low reliability (Table 1). Given that complex mixtures of herbicides are commonly detected in coastal waters, the multisubstance-potentially affected fraction (ms-PAF) method^42^ has been recently applied as a more comprehensive approach to predict the cumulative risk of herbicide mixtures^37^. Although exceedances of WQGVs by individual herbicides are rare, when the combined concentrations of multiple co-occurring herbicides are considered using ms-PAF, exceedances are more frequent^37^. However, the high-reliability WQGVs that are necessary to predict ms-PAFs are not available for most alternative herbicides. Recently, revisions of the WQGVs for 27 GBR-relevant pesticides (including some alternative herbicides) based on all available marine and freshwater toxicity data have been proposed^43,44^; nevertheless, many data gaps remain, especially for marine species. More targeted toxicity testing is therefore warranted to improve current WQGVs for marine species and to develop WQGVs for alternative herbicides where they do not exist.

### Microalgal toxicity tests for derivation of water quality guidelines

National WQGVs (referred to by ANZG^39^ as default GVs) are derived in Australia to protect 99%, 95%, 90% and 80% (PC99, 95, 90, 80, respectively) of marine and freshwater communities by estimating community sensitivity from species sensitivity distributions (SSDs)^45^. The minimum data required for SSDs to meet WQGV criteria are toxicity thresholds for at least five species from at least four phyla that are characteristic of the receiving environment^45^. For a recent and detailed description of the methods and criteria in the Australian context see Warne *et al*.^45^. With rapid growth rates that allow for chronic exposure testing in a short period, marine microalgae represent a suitable taxon to contribute to future SSDs. Currently, SSDs are developed using toxicity data from chronic exposures that are ecologically relevant, and for microalgal toxicity testing inhibition of growth is the most common ecologically relevant endpoint^39,45^. However, strong correlations between effects on microalgae growth and reduced photosynthetic efficiency in estuarine microalgae as measured by Pulse Amplitude Modulation (PAM) fluorometry has been demonstrated for several PSII inhibitor herbicides^23^. The inhibition of effective quantum yield (ΔF/F_m_′) by PSII inhibitor herbicides is proportional to the inhibition of photosynthetic efficiency at a given irradiance^46^ and could be considered as a rapid, sensitive and non-invasive alternative for growth measurements in microalgae toxicity tests involving PSII inhibitor herbicides^23^. In previous studies, inhibition of ΔF/F_m_′ has been extensively applied for assessing the toxicity of PSII inhibitor herbicides in microalgae^23,46–49^ and has also revealed herbicide-induced community tolerance in microalgae to PSII inhibitor herbicides over chronic exposures^50,51^. However, this sensitive photophysiological response may not be suitable as an ecologically relevant measure of whole organism stress for microalgae to non-PSII inhibitor herbicides where the mode of action does not involve PSII^34,52^. Further comparisons between the inhibition of growth and ΔF/F_m_′ as endpoints for herbicide toxicity in marine microalgae are therefore warranted to demonstrate the relevance of using ΔF/F_m_′ as an ecological relevant endpoint in future SSDs.

In order to improve WQGVs for herbicides and expand toxicity threshold data for tropical marine species to alternative herbicides, this study tested the effects of several herbicides on growth and ΔF/F_m_′ to the marine microalgae *Rhodomonas salina*. This species was selected as a tropical representative of an understudied phylum, Cryptophyta, generally underrepresented in SSDs. In addition, this study aimed to derive no effect concentrations (NECs), which are the preferred toxicity estimates for inclusion in SSDs to derive WQGVs. Nine herbicides detected in the GBR and catchments^17,37^ that indicated current toxicity data gaps (based on consultation with the Queensland Department of Environment and Science (DES)) were selected for testing, along with the reference herbicide diuron. The tested herbicides included the PSII inhibitor herbicides tebuthiuron, hexazinone, metribuzin, simazine, propazine, bromacil, and the non-PSII inhibitor herbicides, haloxyfop, 2,4-dichlorophenoxyacetic acid (2,4-D) and imazapic. The toxicity thresholds identified provide valuable toxicity data for alternative herbicides detected in GBR waters and will contribute to new and improved WQGVs for application in risk assessments.

## Results

### Assay performance

*Rhodomonas salina* displayed exponential growth in control treatments across all bioassays with SGR ranging between 1.07 ± 0.07 d^−1^ and 1.29 ± 0.02 d^−1^ (mean ± SD) (Table 2). ΔF/F_m_′ measurements of control treatments varied between 0.45 ± 0.02 and 0.53 ± 0.01 (mean ± SD). The carrier solvents (<0.01% v/v) had no significant influence on SGR compared with filtered seawater after 72 h (ANOVA, F_ethanol_ (1,3) = 1.12; p = 0.37; F_DMSO_ (1,3) = 0.15; p = 0.73). The reference toxicant diuron used in each growth test and fluorescence well plate assay inhibited SGR and ΔF/F_m_′ between 30.1 ± 2.2% and 57.2 ± 2.8% and between 78.4 ± 2.0% and 97.7 ± 2.2% (mean ± SD), respectively (Table 2). This level of variability was expected between independent experiments conducted across 10 occasions and may have been due to minor differences in nutrients or the physiology of cells at the start of each test.

**Table 2:** Assay performance. Specific growth rate (SGR, d^−1^) and photosynthetic efficiency (ΔF/F_m_′) measurements of control and reference (diuron, 4 µg L^−1^) treatments and diuron reference percent inhibition effect (Ref. inh (%)) (mean ± SD; n = 5 per treatment).

Herbicide	SGR d^−1^			ΔF/F_m_′		
	Control	Reference	Ref. inh. (%)	Control	Reference	Ref. Inh. (%)
Diuron	1.20 ± 0.01	0.82 ± 0.03	31.4 ± 2.14	0.53 ± 0.01	0.10 ± 0.01	80.2 ± 1.4
Metribuzin	1.29 ± 0.02	0.55 ± 0.04	57.2 ± 2.8	0.47 ± 0.01	0.03 ± 0.01	93.9 ± 1.4
Hexazinone	1.24 ± 0.04	0.77 ± 0.06	37.7 ± 4.8	0.51 ± 0.01	0.08 ± 0.01	84.8 ± 1.0
Bromacil	1.07 ± 0.06	0.47 ± 0.06	55.9 ± 5.8	0.45 ± 0.02	0.05 ± 0.01	89.0 ± 1.8
Tebuthiuron	1.27 ± 0.02	0.59 ± 0.06	46.4 ± 4.3	0.47 ± 0.01	0.01 ± 0.01	97.7 ± 2.2
Simazine	1.18 ± 0.03	*0.92 ± 0.06	*22.0 ± 5.1	0.49 ± 0.01	0.08 ± 0.01	83.2 ± 0.8
Propazine	1.19 ± 0.02	0.61 ± 0.05	48.3 ± 3.8	0.52 ± 0.01	0.10 ± 0.01	81.4 ± 1.9
Imazapic	1.22 ± 0.03	0.85 ± 0.02	30.1 ± 2.2	0.52 ± 0.01	0.11 ± 0.01	78. 7 ± 1.2
Haloxyfop	1.18 ± 0.05	0.80 ± 0.01	31.8 ± 1.4	0.47 ± 0.01	0.10 ± 0.01	78.4 ± 2.0
2,4 D	1.13 ± 0.07	0.71 ± 0.04	37.0 ± 3.8	0.47 ± 0.01	0.07 ± 0.01	84.5 ± 1.9

*Note for the simazine toxicity bioassay, a reference treatment of diuron, 2 µg L^−1^ was used instead of 4 µg L^−1^ and therefore not included in calculations of the total mean.

Physicochemical measurements indicated little variation within each treatment and across all tests over 72 h: pH 8.5 ± 0.4; salinity 34.2 ± 0.6 PSU, dissolved oxygen 8.0 ± 0.4 mg L^−1^ (± SD, n = 169 for each parameter), temperature 26.0 ± 0.6 °C ( ± SD, 10-min logging intervals). Herbicide concentrations were measured at 0 h and 72 h of each toxicity test to estimate the potential losses of herbicides due to degradation, volatilization or adsorption over the 72-h test duration. Chemical analyses showed that the time-averaged measured concentrations (between 0 h and 72 h samples) were within 20% of nominal concentrations for diuron, metribuzin, hexazinone, bromacil, tebuthiuron, and 2,4-D and between 30-50% of nominal concentrations for propazine, simazine and imazapic. No contaminant was detected in the control treatments and a summary of the nominal and measured concentrations can be found in Table S1.

### Effects of PSII inhibitor herbicides on growth

**Figure 1:**
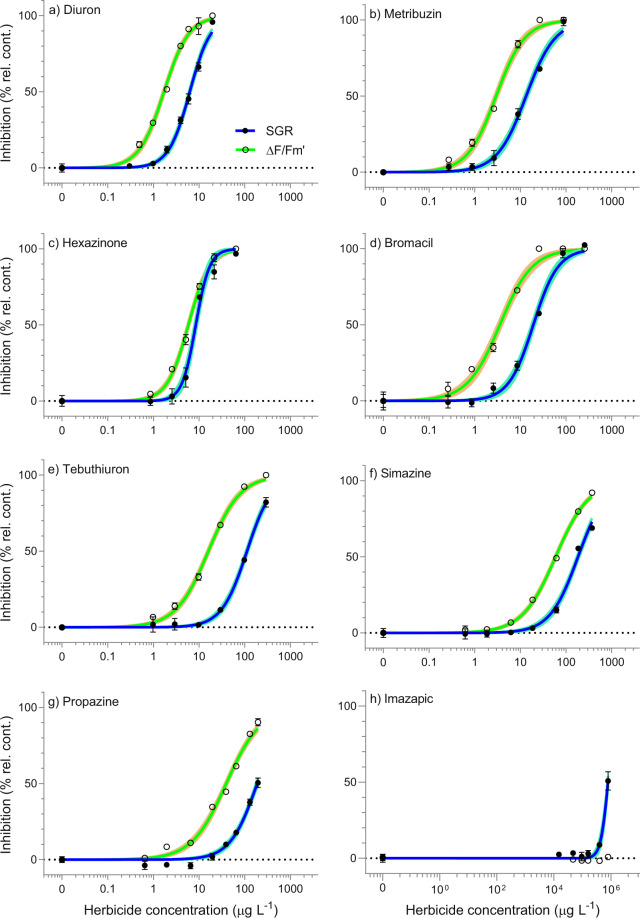
Concentration-response curves for EC_x_ derivation. Sigmoidal, 4-parameter curve fit (solid line) and 95% confidence intervals (shaded area) on the relative percent inhibition of 3-day specific growth rate (SGR; full ring, mean ± SE) and 24 h effective quantum yield (ΔF/F_m_′; open ring, mean ± SE) following herbicide exposure to (**a**) diuron; (**b**) metribuzin; (**c**) hexazinone; (**d**) bromacil; (**e**) tebuthiuron; (**f**) simazine; (**g**) propazine; and (**h**) imazapic at increasing concentrations. All concentrations in µg L^−1^ (n = 5 for each treatment, bars not visible are smaller than symbol).

**Figure 2:**
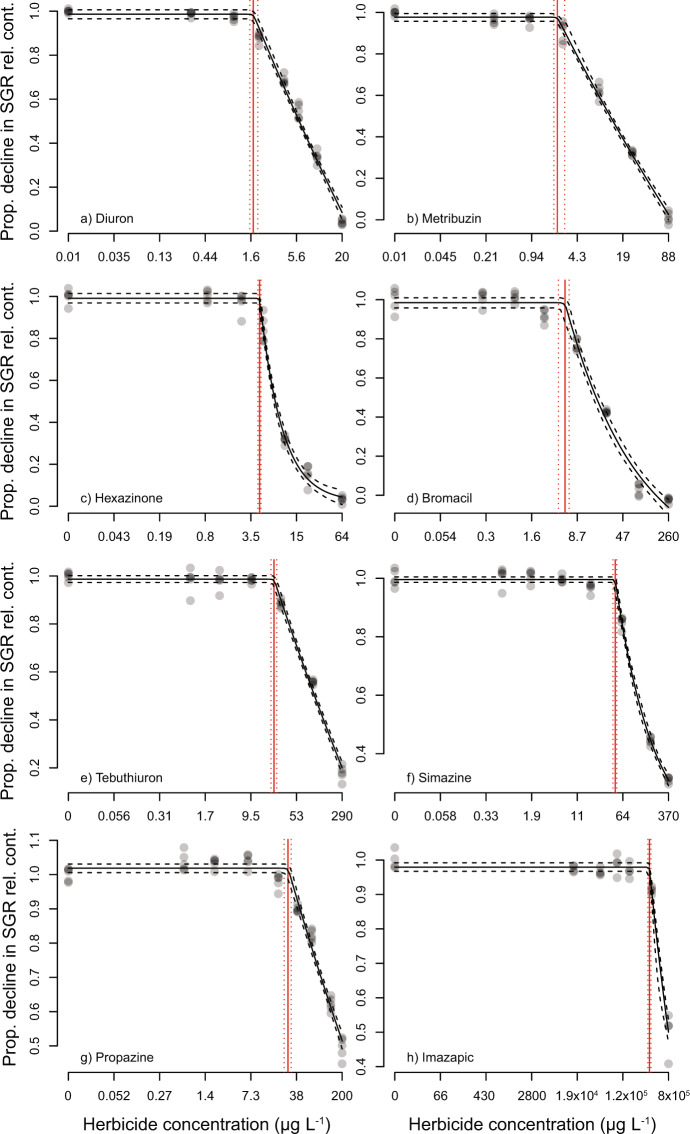
Concentration-response curves for NEC derivation. Bayesian non-linear gaussian model fit on the proportional decline in 3-day specific growth rate (SGR) relative to the control treatment (solid black line) and 95% confidence interval (black dashed line) to derive the no effect concentration (NEC) (red line) and 95% confidence interval (red dashed line) of (**a**) diuron; (**b**) metribuzin; (**c**) hexazinone; (**d**) bromacil; (**e**) tebuthiuron; (**f**) simazine; (**g**) propazine; and h) imazapic. All concentrations in µg L^−1^.

Toxicity tests using *R. salina* were performed on seven PSII inhibitor herbicides, including the reference herbicide diuron (Table 3). The growth of *R. salina* was inhibited by all PSII inhibitor herbicides, and diuron was the most toxic of all PSII inhibitor herbicides with an EC_50_ value of 6.27 µg L^−1^ (Table 3). A summary of the slope and goodness of fit of each concentration-response curve (Sigmoidal, 4 parameter model) for SGR (Fig. 1) is shown in Table S2. The comparison between relative potencies (ReP) based on EC_50_ values to the reference herbicide diuron indicated the order of toxicity: diuron > hexazinone > metribuzin > bromacil > tebuthiuron > simazine > propazine (Table 2). The EC_10_ and modelled no effect concentrations (NECs) were also reported in Table 2 and showed similar orders of toxicity (Fig. 2).

**Table 3:** Toxicity threshold summary. Derived effect concentrations (EC_10_ and EC_50_ from Fig. 1) and no effect concentrations (NECs from Fig. 2) with 95% confidence intervals for each herbicide, and relative equivalent potencies (ReP). NA indicates values could not be calculated. Concentrations are reported in µg L^−1^.

Herbicide	Endpoint	SGR	ΔF/F_m_′	SGR (EC_50_): ΔF/F_m_′ (EC_50_)
Diuron	EC_50_	6.27 (6.02–6.54)	1.71 (1.63–1.80)	3.7
	EC_10_	1.94 (1.75–2.14)	0.43 (0.38–0.48)	
	NEC	1.68 (1.53–1.90)		
	*ReP*	*1*	*1*	
Metribuzin	EC_50_	13.4 (12.3–14.5)	2.95 (2.72–3.18)	4.6
	EC_10_	2.66 (2.21–3.18)	0.60 (0.50–0.71)	
	NEC	2.21 (1.97–2.82)		
	*ReP*	*0.47*	*0.59*	
Hexazinone	EC_50_	8.50 (7.99–9.06)	5.85 (5.61–6.09)	1.5
	EC_10_	3.96 (3.40–4.57)	1.81 (1.63 – 1.99)	
	NEC	4.58 (4.34 – 4.78)		
	*ReP*	*0.71*	*0.29*	
Bromacil	EC_50_	19.3 (17.7–21.0)	3.56 (3.19 – 3.98)	5.4
	EC_10_	4.89 (4.01–5.91)	0.59 (0.45–0.75)	
	NEC	5.53 (4.33 – 6.44)		
	*ReP*	*0.33*	*0.47*	
Tebuthiuron	EC_50_	112 (106–119)	16.0 (15.1–17.0)	7.0
	EC_10_	27.5 (24.2–31.2)	2.66 (2.31–3.06)	
	NEC	22.7 (20.3–25.2)		
	*ReP*	*0.056*	*0.11*	
Simazine	EC_50_	184 (173–195)	59.2 (56.7–61.8)	3.1
	EC_10_	38.4 (33.0–44.2)	9.28 (8.41–10.2)	
	NEC	48.0 (44.0–51.0)		
	*ReP*	*0.034*	*0.029*	
Propazine	EC_50_	188 (177–201)	39.5 (37.1–42.1)	4.8
	EC_10_	42.0 (37.1–47.3)	5.85 (4.90–6.91)	
	NEC	27.8 (24.2 – 31.1)		
	*ReP*	*0.033*	*0.043*	
Imazapic	EC_50_	790,000 (760,000–825,000)	>790,000	NA
	EC_10_	410,000 (362,000–462,000)	>790,000	
	NEC	363,000 (341,000–386,000)		
	*ReP*	*NA*	*NA*	
Haloxyfop	EC_50_	>3,700	>3,700	NA
	EC_10_	>3,700	>3,700	
	NEC	>3,700		
	*ReP*	*NA*	*NA*	
2,4-D	EC_50_	>279,000	>279,000	NA
	EC_10_	>279,000	>279,000	
	NEC	>279,000		
	*ReP*	*NA*	*NA*

### Effects of PSII inhibitor herbicides on effective quantum yield

Diuron, metribuzin, hexazinone, tebuthiuron, and bromacil, caused 100% steady-state inhibition of ΔF/F_m_′ in *R. salina* after 24 h exposures (Fig. 1). Propazine and simazine did not reach 100% steady-state inhibition, peaking at a maximum of 90% inhibition of ΔF/F_m_′ at the highest concentration tested (Fig. 1). A summary of the slope and goodness of fit of each concentration-response curve (Sigmoidal, 4 parameter model) for ΔF/F_m_′ (Fig. 1) is shown in Table S2. The comparison of herbicide concentrations inhibiting ΔF/F_m_′ by 50% (EC_50_) revealed the order of toxicity: diuron > metribuzin > bromacil > hexazinone > tebuthiuron > propazine > simazine (Table 3). Comparable patterns were observed for the order of potencies with respect to ΔF/F_m_′ EC_10_ values (Table 3).

### Toxicity of non-PSII inhibitor herbicides

**Figure 3:**
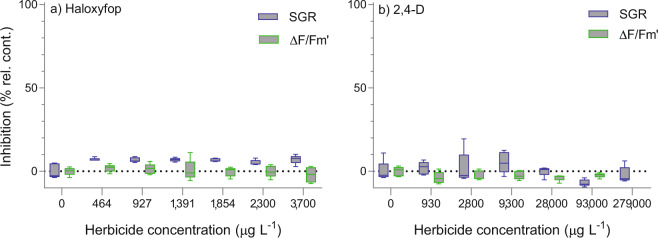
Response of *Rhodomonas salina* to (**a**) haloxyfop and **(b)** 2,4-D. Boxplots showing percent inhibition relative to control treatments in 3-day specific growth rate (SGR d^−1^) and 24 h effective quantum yield (ΔF/F_m_′) (n = 5 for each treatment).

Imazapic inhibited *R. salina* SGR by 50% at a high concentration of 790,000 µg L^−1^, while the same concentration had no effect on ΔF/F_m_′ (F (5,24) = 2.5, p = 0.06) (Fig. 1h, Table 3). Higher concentrations of imazapic caused a decrease in pH to <7.4, therefore effects of imazapic above this concentration were not considered in data analyses. SGR of *R. salina* showed significant differences between control and haloxyfop treatments (F (6,28) = 6.9, p < 0.001); however, inhibition effects across all haloxyfop treatments were consistent (5-7% inhibition) and no relationship between SGR and herbicide concentration between treatments was observed (F (5,24) = 1.1, p = 0.37) (Fig. 3a). ΔF/F_m_′ of *R. salina* was not responsive to haloxyfop (F (6,28) = 0.58, p = 0.74) (Fig. 3a) at the maximum concentration of 3,700 µg L^−1^ (Fig. 3a), which was the highest concentration tested due to its low water solubility. SGR and ΔF/F_m_′ of *R. salina* were nonresponsive to the synthetic auxin-inhibitor 2,4-D at the maximum concentration of 279,000 µg L^−1^ and 93,000 µg L^−1^ (Fig. 3b), respectively tested, and no significant differences between treatments by ANOVA (F (6,28) = 2.2, p = 0.07; F (5,28) = 1.5, p = 0.24, respectively) were detected.

### Relationship between inhibition of effective quantum yield and growth

**Figure 4:**
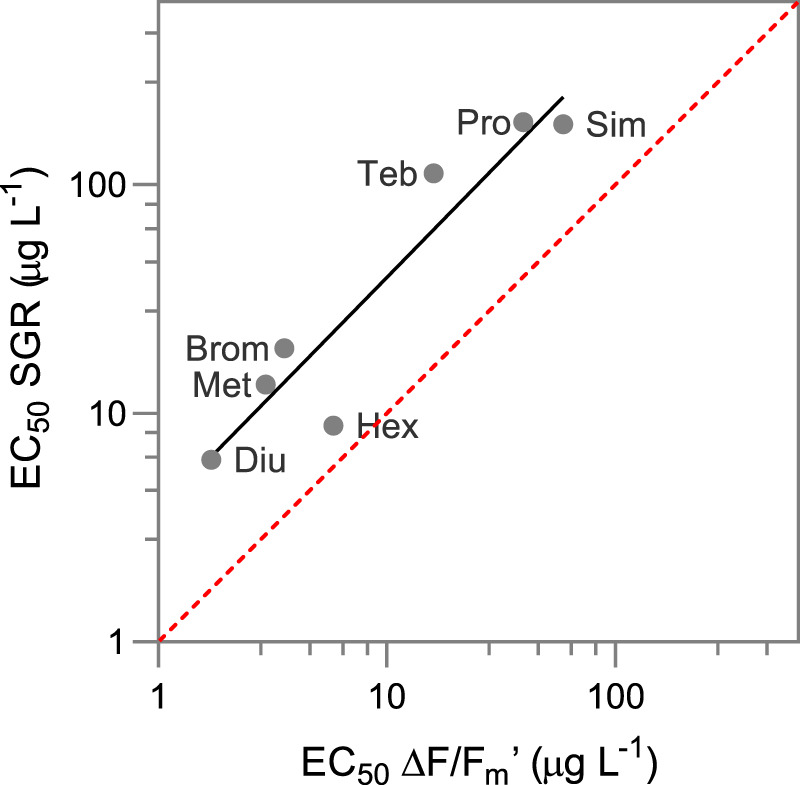
Linear relationship between effective quantum yield (ΔF/F_m_′) and specific growth rate (SGR). Comparison of EC_50_ values (Slope = 3.48; R^2^ = 0.87) of seven PSII inhibitor herbicides (Diu – diuron, Met - metribuzin, Brom - bromacil, Hex - hexazinone, Teb - tebuthiuron, Pro - propazine, Sim - simazine). Dashed red line indicates 1:1 relationship.

The relationship between EC_50_ values for SGR and ΔF/F_m_′ obtained for each PSII herbicide was compared in two ways, with both demonstrating that inhibition of ΔF/F_m_′ was more sensitive than inhibition of growth. Firstly, we compared the EC_50_ ratios for SGR: ΔF/F_m_′ which ranged from 1.5 – 7.0 and averaged 4.3 (Table 3). Secondly, we plotted the linear relationship (R^2^ = 0.87) of EC_50_ values for each herbicide for SGR and ΔF/F_m_′ (Fig. 4) which yielded a slope of 3.48.

## Discussion

### Toxicity effects of PSII inhibitor herbicides

Substantial reductions in both ΔF/F_m_′ and SGR of *R. salina* were observed following exposure to all seven PSII inhibitor herbicides from four different chemical classes. Since Photosystem II is conserved across phototrophs^53^, the response of *R. salina* to the four classes of herbicides including phenylureas (diuron and tebuthiuron), triazines (simazine and propazine), triazinones (metribuzin and hexazinone) and uracils (bromacil) was expected. The toxicities of PSII inhibitor herbicides varied by over 20-fold with respect to inhibition of SGR and ΔF/F_m_′, but a relationship between toxicity and chemical classes was not observed. For example, intra-class variations were wide within the phenylureas with diuron up to 18-times more toxic than tebuthiuron and similar disparities were evident within the triazinones (Table 3). Although all of these herbicides have the same mode of action, differences in PSII activity have been observed in a number of marine phototrophs^21,24,28,47^. Chesworth *et al*.^54^ suggested that herbicides with a greater affinity and faster rate of binding to the Q_B_ site accumulate more effectively leading to higher potencies of these herbicides. Furthermore, the binding of some herbicides to the Q_B_ site lowers the redox potential of the plastoquinone Q_A_/Q_A_- redox couple within PSII, resulting in increased photooxidative stress and subsequently a higher toxicant PSII activity^55,56^, potentially explaining some of the differences observed.

### Comparative species sensitivity

Several studies investigating toxicity of herbicides to tropical marine algae have applied standard test species, such as the diatom *Phaeodactylum tricornutum*^47,49,57^ or symbionts of the family Symbiodiniaceae isolated from corals^21,58^. Toxicity values (EC_10_s and EC_50_s) from these studies and the present study are summarized in Table 4. While some EC_x_ values are similar between species, others differ by up to an order of magnitude (Table 4). Some of these differences will be due to inherent difference is in cell structure, biochemistry and physiology between different species. For example, Millie *et al*.^59^ have shown that algae sensitivity to PSII inhibitor herbicides were related to differences in light-harvesting pigments under different light conditions. Guasch and Sabater^60^ reported that the toxicity of PSII inhibitors was lower for diatom species that were already adapted to low light conditions. In addition, Tang *et al*.^61^ observed higher herbicide sensitivity in chlorophytes compared to diatoms, suggesting some diatoms may apply an extra carbon fixation pathway, such as β-carboxylation that could compensate for the shutdown of PSII-based photosynthesis, and allow algal metabolism to continue^62^. The sensitivity of algal species to PSII inhibitor herbicides has also been shown to be affected by cell size^63^. Although most growth tests are relatively standardized, care should be taken before directly comparing toxicity values between studies, as even subtle differences in experimental exposure and conditions are likely to affect responses.

**Table 4:** Toxicity values for *Rhodomonas salina* and other marine microalgae. Herbicide toxicity to microalgae including data from the United States Environmental Protection Agency (USEPA) ECOTOX Database^94^ and other publications using similar methods as those used in the present study (i.e. experimental conditions, ecological endpoint).

Herbicide	Phyla	Species	Duration	Endpoint growth (µg L^−1^)		Endpoint ΔF/F_m_′ (µg L^−1^)		Reference
				EC_10_	EC_50_	EC_10_	EC_50_	
Metribuzin	Crypto-phyta	*Rhodomonas salina*	3 d; 24 h	2.7	13	0.60	3.0	Present study
	Ochro-phyta	*Skeletonema costatum*	5 d		88			USEPA^94^
Bromacil	Crypto-phyta	*Rhodomonas salina*	3 d; 24 h	4.9	19	0.59	3.6	Present study
	Ochro-phyta	*Skeletonema costatum*	5 d		25			USEPA^94^
Hexazinone	Crypto-phyta	*Rhodomonas salina*	3 d; 24 h	4.0	8.5	1.8	5.9	Present study
	Ochro-phyta	*Navicula sp*.	3 d	6.5	27	3.3	16	Magnusson *et al*.^23^
		*Navicula sp*.	4 h			1.4	5.7	Magnusson *et al*.^47^
		*Phaeodactylum tricornutum*	4 h			1.7	6.6	Magnusson *et al*.^47^
		*Phaeodactylum tricornutum*	2 h			2.9	22	Muller *et al*.^49^
		*Cylindrotheca closteriuma*	4 h			1.7	6.9	Magnusson *et al*.^47^
	Chloro-phyta	*Nephroselmis pyriformis*	3 d	4.8	10	2.1	6.2	Magnusson *et al*.^23^
		*Nephroselmis pyriformis*	4 h			0.47	2.4	Magnusson *et al*.^47^
		*Dunaliella sp*.	24 h				38	Mercurio *et al*.^58^
	Dino-flagellata	*Symbiodinium sp*.	24 h				46	Mercurio *et al*.^58^
		*Symbiodinium sp*.	10 h				8.8	Jones and Kerswell^21^
Tebuthiuron	Crypto-phyta	*Rhodomonas salina*	3 d; 24 h	28	112	2.7	16	Present study
	Ochro-phyta	*Skeletonema costatum*	5 d		60			USEPA^94^
		*Navicula sp*.	4 h			17	94	Magnusson *et al*.^47^
		*Phaeodactylum tricornutum*	4 h			7.6	51	Magnusson *et al*.^47^
		*Cylindrotheca closteriuma*	4 h			10	77	Magnusson *et al*.^47^
	Chloro-phyta	*Nephroselmis pyriformis*	4 h			2.3	12	Magnusson *et al*.^47^
	Dino-flagellata	*Symbiodinium sp*.	10 h				175	Jones and Kerswell^21^
Simazine	Crypto-phyta	*Rhodomonas salina*	3 d; 24 h	38	184	9.3	59	Present study
	Ochro-phyta	*Skeletonema costatum*	5 d		60			USEPA^94^
		*Ceratoneis closterium*	96 h	310				Hook *et al*.^95^
		*Phaeodactylum tricornutum*	3 d	100	580			Osborn and Hook^57^
		*Phaeodactylum tricornutuma*	4 h			11	101	Magnusson *et al*.^47^
		*Phaeodactylum tricornutum*	2 h			18	400	Muller *et al*.^49^
		*Navicula sp*.	4 h			24	157	Magnusson *et al*.^47^
		*Cylindrotheca closteriuma*	4 h			35	242	Magnusson *et al*.^47^
	Chloro-phyta	*Nephroselmis pyriformis*	4 h			3.7	24	Magnusson *et al*.^47^
		*Dunaliella sp*.	24 h				87	Mercurio *et al*.^58^
	Dino-flagellata	*Symbiodinium sp*.	24 h				84	Mercurio *et al*.^58^
		*Symbiodinium sp*.	10 h				150	Jones and Kerswell^21^
Propazine	Crypto-phyta	*Rhodomonas salina*	3 d; 24 h	42	188	5.9	40	Present study
	Ochro-phyta	*Skeletonema costatum*	5 d		25			USEPA^94^
Imazapic	Crypto-phyta	*Rhodomonas salina*	3 d; 24 h	410,000	790,000			Present study
	Ochro-phyta	*Skeletonema costatum*	5 d		<45			USEPA^94^
		*Nephroselmis pyriformis*	3,5,10 d		<1,455			Magnusson *et al*.^69^
		*Navicula sp*.	3,5,10 d		<1,455			Magnusson *et al*.^69^
Haloxyfop	Crypto-phyta	*Rhodomonas salina*	3 d; 24 h	>3,700	>3,700	>3,700	>3,700	Present study
2,4-D	Crypto-phyta	*Rhodomonas salina*	3 d; 24 h	>279,000	>279,000	>93,000	>93,000	Present study
	Ochro-phyta	*Skeletonema costatum*	5 d		>2,000			USEPA^94^
		*Chaetoceros calcitrans*	21 d		9,200			His and Seaman^96^
	Chloro-phyta	*Dunaliella tertiolecta*	1 h				160,220	Mcfeters^97^
		*Dunaliella tertiolecta*	24 h				246,500	Mcfeters^97^

### Relationship between inhibition of effective quantum yield and growth

The inhibition of *R. salina* growth was on average 4-times less sensitive to PSII herbicide exposures than the photoinhibition endpoint (ratios of EC_50_s SGR: ΔF/F_m_′ for each herbicide can be found in Table 2). The correlation plot of EC_50_ values for both endpoints had a slope of 3.5, also showing a greater sensitivity of ΔF/F_m_′ to PSII inhibitor herbicides. Magnusson *et al*.^23^ had demonstrated a relationship between SGR and ΔF/F_m_′ inhibition by PSII inhibitor herbicides that was closer to 1:1 for two tropical benthic microalgae; *Navicula sp*. and *Nephroselmis pyriformis*. Both studies clearly demonstrated a link between inhibition in ΔF/F_m_′ and decreasing growth rates; however, the direct link between the binding of PSII inhibitor herbicides to the D1 protein (reducing electron transport and causing damage to PSII) with growth is not necessarily expected to be 1:1 for all taxa and experimental conditions. Light intensity and light acclimation history have large influences on the relationships between photophysiology, primary production and growth^64^. Furthermore, it has been shown that the pigment structures in some microalgae, such as red algae (rhodophytes) can shift between PSI and PSII, potentially affecting the path of electron transport^65,66^ and direct quantitative links between ΔF/F_m_′, primary production, and SGR may be less certain in rhodophytes than for some other phototrophs. Cryptophytes also contain phycobiliproteins, the characteristic antennae pigments of the prokaryotic cyanobacteria and the eukaryotic rhodophytes^67^. The presence of phycobiliproteins in cryptophytes may allow shifting between PSI and PSII in *R. salina* although, phycobiliproteins are only present in the thylakoid lumen and as phycoerythrin^68^. While Magnusson *et al*.^23^ measured effects on both growth and ΔF/F_m_′ over 3 d, our comparison was between a chronic 3-d growth test and an acute 24-h ΔF/F_m_′ test (as effects of PSII inhibitor herbicides on microalgae typically peak before 6 h and remain consistent over longer periods^69^). While these differences in exposure durations make direct comparisons between the techniques (and against prior studies) more difficult, the exposure durations are optimal for each test type and the linear relationship between effects of multiple PSII inhibitor herbicides on photosynthetic efficiency and growth remains strong. The consistency of these results for a variety of marine microalgae and reported in this study for the marine cryptophyte, in combination with the direct mechanistic link between ΔF/F_m_′ and SGR for PSII inhibitor herbicides, suggests that ΔF/F_m_′ provides a robust and sensitive endpoint for determining sub-lethal effect thresholds for these herbicides.

### Toxicity effects of non-PSII inhibitor herbicides

*R. salina* was far more sensitive to the PSII inhibitor herbicides than the non-PSII inhibitor herbicides tested here. *R. salina* was insensitive to non-PSII inhibitor herbicides within the phenoxy family, haloxyfop and 2,4-D at the highest concentrations tested. Growth regulator herbicides, such as 2,4-D, inhibit the plant hormone auxin and are primarily used as selective herbicides for controlling broadleaves (dicots)^43^. This pathway is not present in cryptophytes, explaining the lack of toxicity of 2,4-D to *R. salina*. Previous studies reported similar observations of the low toxicity of 2,4-D on the growth rate of marine microalgae (Table 4), with the most sensitive species the diatom *Chaetoceros calcitrans* (21 d, EC_50_ = 9.2 mg L^−1^). *R. salina* was also less responsive to haloxyfop, which targets the acetyl-CoA carboxylase enzyme involved in the synthesis of lipids and fatty acids in plants^44^. ACCase inhibitors target the homomeric (eukaryotic) form of the enzyme rather than the heteromeric (prokaryotic) form^44^ and microalgae, such as rhodophytes and chlorophytes contain the heteromeric ACCase enzyme in their plastids^70^, likely explaining the insensitivity towards haloxyfop in cryptophytes. Imazapic was only toxic to *R. salina* at high concentrations. Imazapic inhibits the activity of the enzyme acetohydroxy acid synthase (AHAS or ALS), which is responsible for catalyzing the production of several branched-chain aliphatic amino acids across many aquatic phototrophs^71^. Other marine microalgae are similarly insensitive to imazapic, and the sensitivity to imazapic of enzyme variants in marine microalgae are unknown. No effect on the growth rates of the marine microalgae *Navicula sp*. and *Nephroselmis pyriformis* were observed after 10 d exposure at concentrations of up to 1.5 mg L^−1^ ^69^. Conversely, imazapic is far more toxic to freshwater phototrophs. For example, imazapic has an EC_50_ of 6.1 µg L^−1^ for growth in the freshwater macrophyte *Lemna gibba* (duckweed)^72^. In macrophytes, imazapic is absorbed through the roots and shoots of plants, possibly explaining the lower toxicity of imazapic to microalgae^71^. Another factor to consider with respect to the sensitivity of marine species is whether the structure of imazapic may affect its exposure and bioavailability in seawater. Imazapic contains a carboxylic acid (COOH) which may result in complexation with Mg^2+^ and Ca^2+^ ions in the seawater^73^, or stabilize the herbicide at the seawater:air interface^74^. Both mechanisms could reduce the exposure and bioavailability of imazapic to marine species accounting for the low toxicities reported.

### Toxicity thresholds for guideline development

Water quality guidelines are usually developed using SSDs^75^ and ideally from NEC or EC_10_ values for multiple diverse taxa^45^. However, most current herbicide WQGVs for marine communities are of low reliability (e.g. developed from toxicity data for as few as five species), or have not yet been developed due to the lack of data^39^. Currently, WQGVs exist only for the five priority herbicides (diuron, atrazine, ametryn, tebuthiuron, hexazinone) and four alternative herbicides (bromacil, MCPA, simazine, and 2,4-D)^39^ (Table 1). There are no WQGVs for metribuzin, propazine, haloxyfop, and imazapic. A comparison of the existing ANZG WQGVs^39^ and proposed guideline values (PGVs)^43,44^ against herbicide toxicity thresholds (SGR: NEC and EC_10_ values) for *R. salina* is presented in Table 1. The NEC and EC_10_ values for hexazinone and bromacil were far lower than current PC99 WQGVs; however, the PC99 PGVs would all be protective of *R. salina* (Table 1). Nevertheless, most of the PGVs are of very low to moderate reliabiltiy^43,44^ (Table 1) and could be improved by the incorporation of toxicity data from additional species, such as *R. salina*. Apart from diuron (the most toxic herbicide tested in this study), the NEC and EC_10_ values were all greater than 2 µg L^−1^ and above concentrations that have been detected in tropical coastal waters^13,16,17,37^. However, the risks posed by these herbicides should not be assessed individually as they are usually detected in complex mixtures of multiple herbicides. Instead, their contribution to the total risk can be assessed using ms-PAF^42^, which accounts for all herbicides that have reliable SSDs (and WQGVs). The ms-PAF method has also been extended to include the additional influence of heatwave conditions on WQGVs for pesticides^76^. The exceedance of PC99 values for herbicide mixtures has recently been reported in water quality monitoring programs using ms-PAF, where individual herbicide did not exceed their own PC99 values^37^. The development of SSDs for alternative herbicides detected in the GBR using relevant toxicity data (such as the *R. salina* data presented here) will allow their contribution in predicting the cumulative risks of herbicide mixtures using ms-PAF.

## Conclusion

Alternative herbicides may be practical substitutes for controlling weeds; however, their toxicity to non-target species such as *R. salina* could contribute to the combined risks posed by herbicide mixtures regularly detected in coastal waters in the tropics. In the present study, exposures of *R. salina* to increasing herbicide concentrations resulted in inhibition of ΔF/Fm′ within 24 h, indicating reduced photosynthetic efficiency which led to reduced growth rates over 72 h chronic exposures. Photoinhibition was a more sensitive endpoint over 24 h than inhibition of growth over 72 h; however, the relationship between inhibition of ΔF/Fm′ and SGR was linear and consistent. Importantly, the non-PSII inhibitor herbicides (imazapic, 2,4-D, haloxyfop) were substantially less toxic than the most toxic PSII inhibitor herbicides, indicating these herbicides pose little risk to this microalga in the marine environment. The toxicity thresholds (NECs and EC10s) derived here were higher than concentrations detected in tropical marine waters. However, the risk posed by these herbicides to marine species is better assessed by comparing measured values in the field against high-reliability WQGVs that are derived from SSDs. The current study contributes targeted data towards developing SSDs for alternative herbicides that are essential to improve predictions of the cumulative ecological risks posed by herbicide mixtures (using ms-PAF) detected in marine monitoring programs. While this study targeted some of the most frequently detected alternative herbicides in GBR waters, there remains a number of pesticides, including insecticides and fungicides with no current WQGVs and further testing is needed to address this.

## Methods

### Test species and culture conditions

The cryptophyte *Rhodomonas salina* (Wislouch)^77^ (CS 24/01) was purchased from the Australian National Algae Supply Service, Hobart (CSIRO). Cryptophytes are an important component of the primary producers in both freshwater and marine habitats, and changes in their abundance, composition and nutritional value may initiate an indirect bottom-up effect on higher trophic levels^78^. Many species are widespread and abundant in the sea within wide temperature ranges (5–29 °C), which make this phylum highly suitable for acute and chronic toxicity tests in a short period of time under both temperate and tropical conditions^79–81^. Cultures of *R. salina* were established three weeks prior to experimentation in Guillard’s f_2_ marine medium (0.5 mL of AlgaBoost F/2, AusAqua in 1 L sterile 0.5 µm-filtered seawater (FSW; pH 8.0, salinity 35.0 psu))^82^. Cultures were maintained in sterile 500 mL Erlenmeyer flasks as batch cultures in exponential growth phase with twice-weekly transfers of 70 mL of a 3- to 4-day-old *R. salina* suspension to 300 mL f_2_ medium under sterile conditions. Clean culture solutions were aerated and maintained at 26 ± 1 °C and under a 12:12 h light:dark cycle (90-100 μmol photons m^–2^ s^–1^, Osram Lumilux Cool White 36 W).

### Herbicide stock preparation

Herbicide stock solutions were prepared using PESTANAL (Sigma-Aldrich) analytical grade products (HPLC ≥ 98%): diuron (CAS 330-54-1), metribuzin (CAS 21087-64-9), hexazinone (CAS 51235-04-2), tebuthiuron (CAS 34014-18-1), bromacil (CAS 314-40-9), propazine (CAS 139-40-2), simazine (CAS 122-34-9), imazapic (CAS 104098-48-8), haloxyfop (CAS 72619-32-0), 2,4-D (CAS 94-75-7). The selection of herbicides was based on consultation with the Queensland DES and detection frequency in coastal waters of the GBR^17,37^. Stock solutions were prepared in sterile 1 L Schott glass bottles using ultra-pure water (milli-Q, Millipore) or sterile 0.5 µm-FSW. Diuron and simazine were dissolved using HPLC-grade ethanol (<0.001% (v/v) in exposures). Haloxyfop was dissolved in dimethyl sulfoxide (DMSO) (≤ 0.006% (v/v) in exposure). No solvent carrier was used for the preparation of the remaining herbicide stock solutions. A summary of herbicide stocks, solvent carriers, nominal and measured concentrations, as well as chemical properties of the tested herbicides can be found in Table S1.

### Toxicity test protocol

Cultures of *R. salina* were exposed to a range of herbicide concentrations over a period of 72 h. Inoculum was taken from cultures in the exponential growth phase (4-day-old with cell density of approximately 1 × 10^6^ cell mL^−1^). Prior to the inoculation of the test solutions, 15 mL of algae suspension (of the 4-day-old algal culture) was washed in 30 mL sterile 0.5 µm-FSW by centrifuge in 50 mL Falcon tubes at 1500 *g* for 5 minutes (Eppendorf Centrifuge 5810 R, Bio-strategy). The supernatant was decanted, and the cell pellet re-suspended in 30 mL of sterile 0.5 µm-FSW and homogenized by vortexing. This process was repeated three times to remove the nutrient-enriched f_2_ culture medium, which might affect herbicide toxicity^83–86^. The cell pellet was finally re-suspended in about 15 mL of sterile 0.5 µm-FSW. The cell density of the concentrated algae suspension was measured from two 500 µL sub-samples by flow cytometry. The desired inoculum was calculated to a given starting cell density of 3 × 10^3^ cells mL^−1^ in the following toxicity test. Individual *R. salina* working suspensions for each herbicide treatment were prepared in individual 100 mL Schott glass bottles by adding the required algae inoculum and sterile 0.5 µm-FSW. Each Schott glass bottle was finally dosed with a range of herbicide concentrations (Table S1). Bioassays for each herbicide were performed on different days with fresh algae, FSW and herbicide stocks. In each bioassay, a control (no herbicide) and reference (diuron, 4 µg L^−1^) treatment was added to ensure the response is reproducible. Diuron was chosen as a reference toxicant as it is a widespread contaminant and its toxic mode of action (PSII inhibition) and toxicity to a wide variety of microalgae are well understood (see Magnusson *et al*.^23^).

Five replicated aliquots of 10 mL were transferred from the individual 100 mL Schott glass bottles into sterile 20 mL glass scintillation vials and incubated at 26.0 ± 0.6 °C under a 12:12 h light:dark cycle at 90-100 μmol photons m^–2^ s^–1^ (Osram Lumilux Cool White 36 W). Vials were randomized and swirled daily. Sub-samples of 500 µL were taken from each replicate to measure cell densities of algal populations at 0 h and 72 h using a flow cytometer (BD Accuri C6, BD Biosciences, CA, USA) equipped with red and blue lasers (14.7 mW 640 nm Diode Red Laser 20 mW 488 nm Solid State Blue Laser) and standard filter setup. The flow rate was set to 35 µL min^−1^, 16-µm core size with a sample volume of 50 µL. Cell densities were obtained by plotting a two-dimensional cytogram. A fixed gating was used around the viable (chlorophyll fluorescing) cells, which allowed for differentiation of non-algal particles (debris) and dead cells from viable *R. salina* cells. Viable cells typically represented 75 - 95% of particles counted (control treatment at 72 h). Each 500 µL sub-sample was analyzed by the flow cytometer two times and an average taken of the number of events that occur within the gated region. This process was then repeated for each replicate per treatment. Specific growth rates (SGR) were expressed as the logarithmic increase in cell density from day i (t_i_) to day j (t_j_) as per Eq. (1), where SGR_i-j_ is the specific growth rate from time i to j; X_j_ is the cell density at day j and X_i_ is the cell density at day i^87^.

1 $${{\rm{SGR}}}_{{\rm{i}}-{\rm{j}}}=\frac{\mathrm{ln}\,{{\rm{X}}}_{{\rm{j}}}-\,\mathrm{ln}\,{{\rm{X}}}_{i}}{{{\rm{t}}}_{{\rm{j}}}-{{\rm{t}}}_{{\rm{i}}}}\,{({\rm{day}}}^{-1})$$

SGR relative to the control treatment was used to derive chronic effect values for growth inhibition. A test was considered valid if the SGR of control replicates was ≥0.92 day^−1^ ^87^.

### Chlorophyll fluorescence measurements

Acute effects of herbicides on the photophysiology of *R. salina*, measured by chlorophyll fluorescence as the effective quantum yield (ΔF/F_m_′), were investigated in non-pyrogenic polystyrene 48 well-plates with lid (Nunclon Delta, Thermo Scientific) using imaging PAM fluorometry (I-PAM, Walz, Germany)^46,88^, following an exposure period of 24 h at an irradiance of 90-100 μmol photons m^–2^ s^–1^. Inoculum was taken from mother cultures in the exponential growth phase (4-day-old with cell density of approximately 1 × 10^6^ cell mL^−1^). Initial testing of varying cell densities indicated that consistent ΔF/F_m_′ measurement signals >0.45^46^ were obtained at a starting cell density of 3.5 × 10^5^ cells mL^−1^ (equivalent to cell density after ~3 d in the SGR inhibition test). Individual *R. salina* working suspensions for each herbicide treatment were prepared in individual 50 mL Schott glass bottles by adding algae inoculum and sterile f_2_ (0.5 µm-FSW) marine medium. Each 50 mL Schott glass bottle was finally dosed with a range of herbicide concentrations (Table S1). Five replicated aliquots of 1 mL were transferred from the individual 50 mL Schott glass bottles across two 48-well plates (randomly) and incubated at 26.0 ± 0.6 °C under a 12:12 h light:dark cycle at 90-100 μmol photons m^–2^ s^–1^ (Osram Lumilux Cool White 36 W). Replicated seawater controls (SWC) (n = 5) or solvent controls (SC) and diuron references (4 µg L^−1^) were included randomly across each 48-well plate to ensure consistency in inhibition response between replicated algae cultures. Light adapted minimum fluorescence (F) and maximum fluorescence (F_m_′) were determined and effective quantum yield was calculated for each treatment as per Eq. (2) ^88^. The timing of plate preparation and measurements were staggered to ensure a consistent exposure duration of 24 h. Imaging PAM settings were set to actinic light = 1 (corresponding to photosynthetically active radiation (PAR) of 90-100 μmol photons m^−2^ s^−1^), measuring intensity = 11, gain = 3; damp = 2.

2 $$\frac{\Delta {\rm{F}}}{{\rm{F}}{\rm{m}}{\prime} }\,=\,\frac{{\rm{F}}{\rm{m}}{\prime} \mbox{--}{\rm{F}}}{{\rm{F}}{\rm{m}}{\prime} }$$

A screening process of plates containing algae suspension only was performed immediately prior to exposure with herbicides to ensure that ΔF/F_m_′ > 0.45.

### Physicochemical analyses

Physico-chemical water quality parameters including pH and salinity (LAQUAact-PC110 Meter, HORIBA Scientific) and dissolved oxygen (HQ30D Portable Meter, HACH) were measured from individual 100 mL Schott glass bottles at 0 h and replicated 20 mL glass scintillation vials pooled for each concentration at 72 h. Temperature was logged in 10-min intervals over the total test duration (HOBO, Onset). Analytical samples were also taken from individual 100 mL Schott glass bottles at 0 h and replicated 20 mL glass scintillation vials pooled for each concentration at 72 h. Aliquots (1 mL) were transferred into 1.5 mL Liquid Chromatography amber glass vials and spiked with surrogate standards (i.e. diuron-D6, hexazinone-D6, metribuzin-D3, simazine-D10, propazine-D6, bromacil-D3, haloxyfop-D4, 2,4-D-^13^C_6_, and imazapic-D7) at a final concentration of 10 ng mL^−1^. Prior to analysis samples were stored at −20 °C, defrosted and centrifuged. Herbicide concentrations were determined by HPLC-MS/MS using a SCIEX Triple Quad 6500 QTRAP mass spectrometer (SCIEX, Concord, Ontario, Canada) equipped with a TurboIonSpray probe^10,89,90^. The mass spectrometer was coupled to a Shimadzu Nexera X2 uHPLC system (Shimadzu Corp., Kyoto, Japan) using a Phenomenex Kinetex Biphenyl column (2.6 μm 50 ×2.1 mm 100 Å) for analyte separation. Five μL of sample was injected on to the column followed by a linear gradient starting at 10% B for 0.5 min, ramped to 100% B in 4.7 min then held at 100% for 4.0 min followed by equilibration at 10% B for 3.0 min (A = 1% methanol in milli-Q water, B = 95% methanol in milli-Q water, both containing 0.1% acetic acid). The mass spectrometer was operated in both positive and negative ion mode using a scheduled multiple reaction-monitoring method (sMRM). Positive samples were confirmed by retention time and by comparing transition intensity ratios between the sample and an appropriate calibration standard from the same run. To provide estimates of ‘measured’ concentrations used for concentration-response modelling the geometric mean from measured start and end concentrations (time-weighted average) was assigned as the ‘actual’ concentration in that sample. The average loss from these measured concentrations was then applied to all nominal concentrations.

### Statistical analyses

All statistical analyses were based on measured herbicide concentrations. Mean percent inhibition in SGR and ΔF/F_m_′ of each treatment relative to the control treatment was calculated as per Eq. (3) ^87^, where X_control_ is the average SGR or ΔF/F_m_′ of control and X_treatment_ is the average SGR or ΔF/F_m_′ of single treatments.

3 $$ \% \,{\rm{Inhibition}}=\frac{{{\rm{X}}}_{{\rm{control}}}-{{\rm{X}}}_{{\rm{treatment}}}}{{{\rm{X}}}_{{\rm{control}}}}\times 100$$

Nonlinear regression (Sigmoidal, 4-parameter) was used to produce concentration-response curves for each herbicide test (GraphPad Prism V 8.0.). Effective concentrations inhibiting ΔF/F_m_′ and SGR by 10% and 50% with 95% confidence intervals (EC_10_/EC_50_) relative to the control were interpolated from the equations of the curve fit. One-factor analysis of variance (ANOVA with replicates) was used to determine if there were significant differences (p < 0.05) in algal SGR rates and ΔF/F_m_′ samples between various herbicide treatments. The relative potencies of the herbicides were determined using the relative equivalent potencies (ReP) compared to the reference herbicide diuron (EC_50_ diuron/EC_50_ herbicide)^23^. ReP values > 1 indicate potencies proportionally greater than diuron and ReP values < 1 indicate potencies less than diuron.

The estimation of no effect concentrations (NEC) was calculated in R (Version 3.6.1). The proportional decline in SGR (1-inhibition) was modelled as a function of log concentration of each herbicide using a Bayesian non-linear gaussian model using the R package jagsNEC^91^. This model has been specifically developed to derive no effect concentrations (NECs) but also allows the estimation of EC_10_ and EC_50_ values and is adapted from Fox^92^, and more generally defined by Eq. (4) ^92^:

4 $${\rm{E}}\,[{{\rm{Y}}}_{i}{|{\rm{x}}}_{{\rm{i}}}]={{\rm{\mu }}}_{{\rm{i}}}={\rm{\alpha }}\,\exp [-{\rm{\beta }}({{\rm{x}}}_{{\rm{i}}}-{\rm{\gamma }}){\rm I}({{\rm{x}}}_{{\rm{i}}}-{\rm{\gamma }})]-\Delta $$

E[Y_i_|x_i_] is the mathematical expectation of Y_i_ (the response, e.g. in this case, the proportional decline in SGR) conditional on a given concentration x_i_. The model parameters for the generalized case are *α* (the response at zero or low concentrations, also called ‘top’), −*β* (the rate of decay in the response after the NEC) and γ (the NEC value)^92^. For a gaussian *Y*, as used here, the model has the additional parameters Δ (an offset or intercept) and σ (the random error variance in *Y*). We used un-informative priors for the model parameters, including: *α* ~ dnorm(0, 0.1), β ~ dgamma(0.0001,0.0001), *γ* ~ dnorm(0, 0.01), Δ ~ dnorm(0, 0.1), and σ ~dunif(0, 29). Note that in jags dnorm is parameterized as a mean and precision (rather than mean and SD, as in R). Models were run with 10,000 Markov chain Monte Carlo (MCMC) iterations after an initial ‘burn-in’ period of 20000 iterations and for five separate chains. Trace plots were used to evaluate model fits and were found to have relatively good mixing in all cases.

## Supplementary information

Supplementary information.

## References

[1] Basheer C, Obbard JP, Lee HK (2003). Persistent organic pollutants in Singapore’s coastal marine environment: part I, seawater. Water, Air, Soil Pollut..

[2] Ali HR (2014). Contamination of diuron in coastal waters around Malaysian Peninsular. Mar. Pollut. Bull..

[3] Okamura H, Aoyama I, Ono Y, Nishida T (2003). Antifouling herbicides in the coastal waters of western Japan. Mar. Pollut. Bull..

[4] Roche, H., Salvat, B. & Ramade, F. Assessment of the pesticides pollution of coral reefs communities from French Polynesia. *Rev. Ecol*. (2011).

[5] Sarkar SK (2008). Occurrence, distribution and possible sources of organochlorine pesticide residues in tropical coastal environment of India: an overview. Environ. Int..

[6] Castillo LE, de la Cruz E, Ruepert C (1997). Ecotoxicology and pesticides in tropical aquatic ecosystems of Central America. Environ. Toxicol. Chem..

[7] Hernández-Romero AH, Tovilla-Hernández C, Malo EA, Bello-Mendoza R (2004). Water quality and presence of pesticides in a tropical coastal wetland in southern Mexico. Mar. Pollut. Bull..

[8] Carbery K, Owen R, Frickers T, Otero E, Readman J (2006). Contamination of Caribbean coastal waters by the antifouling herbicide Irgarol 1051. Mar. Pollut. Bull..

[9] Kennedy K (2012). The influence of a season of extreme wet weather events on exposure of the World Heritage Area Great Barrier Reef to pesticides. Mar. Pollut. Bull..

[10] Mercurio P (2016). Degradation of herbicides in the tropical marine environment: Influence of light and sediment. Plos One.

[11] Lewis, S. E. *et al*. Using monitoring data to model herbicides exported to the Great Barrier Reef, Australia. In: The 19th International Congress on Modelling and Simulation. Modelling and Simulation Society of Australia and New Zealand. *MODSIM2011*, 2051–2056 (2011).

[12] Shaw M (2010). Monitoring pesticides in the Great Barrier Reef. Mar. Pollut. Bull..

[13] Grant, S. *et al*. Marine Monitoring Program: Annual Report for inshore pesticide monitoring 2015–2016. Report for the Great Barrier Reef Marine Park Authority, Great Barrier Reef Marine Park Authority, Townsville, Australia. (2017).

[14] Devlin, M. M. *et al*. Advancing our understanding of the source, management, transport and impacts of pesticides on the Great Barrier Reef 2011–2015. Report for the Queensland Department of Environment and Heritage Protection. Tropical Water & Aquatic Ecosystem Research (TropWATER) Publication, James Cook University, Cairns, Australia. (2015).

[15] GBRMPA. Reef 2050 integrated monitoring and reporting program: strategy update 2018. Great Barrier Reef Marine Park Authority, Townsville, Australia, http://www.environment.gov.au/marine/gbr/publications/reef-2050-long-term-sustainability-plan-2018 (2018).

[16] Lewis SE (2009). Herbicides: a new threat to the Great Barrier Reef. Environ. Pollut..

[17] O’Brien D (2016). Spatial and temporal variability in pesticide exposure downstream of a heavily irrigated cropping area: application of different monitoring techniques. J. Agric. Food Chem..

[18] Radcliffe, J. Pesticide use in Australia. A review undertaken by the Australian Academy of Technological Sciences, Victoria, Australia, https://www.atse.org.au/ (2002).

[19] Oettmeier, W. Herbicides of photosystems II. In: Barber, J., 1992. Structure, Function and Molecular Biology. *Elsevier*, 349–408, 10.1016/B978-0-444-89440-3.50018-7 (1992).

[20] Negri A (2005). Effects of the herbicide diuron on the early life history stages of coral. Mar. Pollut. Bull..

[21] Jones RJ, Kerswell AP (2003). Phytotoxicity of photosystem II (PSII) herbicides to coral. Mar. Ecol. Prog. Ser..

[22] Bengston-Nash SM, Quayle PA, Schreiber U, Muller JF (2005). The selection of a model microalgal species as biomaterial for a novel aquatic phytotoxicity assay. Aquat. Toxicol..

[23] Magnusson M, Heimann K, Negri AP (2008). Comparative effects of herbicides on photosynthesis and growth of tropical estuarine microalgae. Mar. Pollut. Bull..

[24] Negri AP, Flores F, Röthig T, Uthicke S (2011). Herbicides increase the vulnerability of corals to rising sea surface temperature. Limnol. Oceanogr..

[25] van Dam JW, Negri AP, Mueller JF, Uthicke S (2012). Symbiont-specific responses in foraminifera to the herbicide diuron. Mar. Pollut. Bull..

[26] Haynes D, Ralph P, Prange J, Dennison B (2000). The impact of the herbicide diuron on photosynthesis in three species of tropical seagrass. Mar. Pollut. Bull..

[27] Flores, F., Collier, C. J., Mercurio, P. & Negri, A. P. Phytotoxicity of four photosystem II herbicides to tropical seagrasses. *Plos One* **8**, 10.1371/journal.pone.0075798 (2013). 10.1371/journal.pone.0075798 PMC3786934 24098726

[28] Wilkinson AD, Collier CJ, Flores F, Negri AP (2015). Acute and additive toxicity of ten photosystem-II herbicides to seagrass. Sci. Rep.

[29] Ralph PJ (2000). Herbicide toxicity of *Halophila ovalis* assessed by chlorophyll a fluorescence. Aquat. Bot..

[30] Negri AP, Flores F, Mercurio P, Mueller JF, Collier CJ (2015). Lethal and sub-lethal chronic effects of the herbicide diuron on seagrass. Aquat. Toxicol..

[31] Cantin NE, Negri AP, Willis BL (2007). Photoinhibition from chronic herbicide exposure reduces reproductive output of reef-building corals. Mar. Ecol. Prog. Ser..

[32] King J, Alexander F, Brodie J (2013). Regulation of pesticides in Australia: the Great Barrier Reef as a case study for evaluating effectiveness. Agric., Ecosyst. Environ.

[33] Davis A, Lewis S, Brodie J, Benson A (2014). The potential benefits of herbicide regulation: a cautionary note for the Great Barrier Reef catchment area. Sci. Total Environ..

[34] Duggleby RG, McCourt JA, Guddat LW (2008). Structure and mechanism of inhibition of plant acetohydroxyacid synthase. Plant Physiol. Biochem..

[35] Shaner DL, Anderson PC, Stidham MA (1984). Imidazolinones: potent inhibitors of acetohydroxyacid synthase. Plant Physiol..

[36] Secor J, Cséke C (1988). Inhibition of acetyl-CoA carboxylase activity by haloxyfop and tralkoxydim. Plant Physiol..

[37] Gallen, C. *et al*. Marine Monitoring Program: Annual Report for inshore pesticide monitoring 2017–18. Report for the Great Barrier Reef Marine Park Authority, Great Barrier Reef Marine Park Authority, Townsville, 118 pp, http://elibrary.gbrmpa.gov.au/jspui/handle/11017/3489 (2019).

[38] Brodie J (2012). Terrestrial pollutant runoff to the Great Barrier Reef: an update of issues, priorities and management responses. Mar. Pollut. Bull..

[39] ANZG. Revised Australian and New Zealand guidelines for fresh and marine water quality. Australian and New Zealand Environment and Conservation Council and Agriculture and Resource Management Council of Australia and New Zealand, Canberra, Australia, https://www.waterquality.gov.au/anz-guidelines/guideline-values/default/water-quality-toxicants/toxicants (accessed 10 March 2020) (2018).

[40] CCME. Canadian water quality guidelines for the protection of aquatic life. Canadian Council of Ministers of the Environment, Winnipeg, Canada, https://www.ccme.ca/en/resources/canadian_environmental_quality_guidelines/index.html (accessed 26 May 2019) (2014).

[41] EU. Directive 2013/39/EU of the european parliament and of the council of 12 August 2013 amending directives 2000/60/EC and 2008/105/EC as regards priority substances in the field of water policy. *Official J. Eur. Union* 226, http://data.europa.eu/eli/dir/2013/2039/oj (2013).

[42] Traas, T. P. *et al*. The potentially affected fraction as a measure of ecological risk in Posthuma, L., Suter, G. W. II, Traas, T. P., eds, Species Sensitivity Distributions in Ecotoxicology. Lewis, Boca Raton, FL, USA, pp 315–344. (2002).

[43] King, O., Smith, R., Mann, R. & Warne, M. St. J. Proposed aquatic ecosystem protection guideline values for pesticides commonly used in the Great Barrier Reef catchment area: Part 1 (amended) - 2,4-D, Ametryn, Diuron, Glyphosate, Hexazinone, Imazapic, Imidacloprid, Isoxaflutole, Metolachlor, Metribuzin, Metsulfuron-methyl, Simazine, Tebuthiuron. Department of Environment and Science, Brisbane, Australia. 296 pp, https://www.publications.qld.gov.au/dataset/proposed-guideline-values-27-pesticides-used-in-the-gbr-catchment (2017).

[44] King, O., Smith, R., Warne, M. St. J. & Mann, R. Proposed aquatic ecosystem protection guideline values for pesticides commonly used in the Great Barrier Reef catchment area: Part 2 - Bromacil, Chlorothalonil, Fipronil, Fluometuron, Fluroxypyr, Haloxyfop, MCPA, Pendimethalin, Prometryn, Propazine, Propiconazole, Terbutryn, Triclopyr and Terbuthylazine. Department of Science, Information Technology and Innovation, Brisbane, Australia. 211 pp, https://www.publications.qld.gov.au/dataset/proposed-guideline-values-27-pesticides-used-in-the-gbr-catchment (2017).

[45] Warne, M. St. J. *et al*. Revised Method for Deriving Australian and New Zealand Water Quality Guideline Values for Toxicants – update of 2015 version. Prepared for the revision of the Australian and New Zealand Guidelines for Fresh and Marine Water Quality. Australian and New Zealand Governments and Australian state and territory governments, Canberra, Australia. 48 pp, https://www.waterquality.gov.au/anz-guidelines/guideline-values/derive/warne-method-derive, 10.13140/RG.2.2.36577.35686 (2018).

[46] Schreiber U, Quayle P, Schmidt S, Escher BI, Mueller JF (2007). Methodology and evaluation of a highly sensitive algae toxicity test based on multiwell chlorophyll fluorescence imaging. Biosens. Bioelectron.

[47] Magnusson M, Heimann K, Quayle P, Negri AP (2010). Additive toxicity of herbicide mixtures and comparative sensitivity of tropical benthic microalgae. Mar. Pollut. Bull..

[48] Bengston-Nash SM, Schreiber U, Ralph PJ, Muller JF (2005). The combined SPE: ToxY-PAM phytotoxicity assay; application and appraisal of a novel biomonitoring tool for the aquatic environment. Biosens. Bioelectron.

[49] Muller R (2008). Rapid exposure assessment of PSII herbicides in surface water using a novel chlorophyll a fluorescence imaging assay. Sci. Total Environ..

[50] Magnusson M, Heimann K, Ridd M, Negri AP (2012). Chronic herbicide exposures affect the sensitivity and community structure of tropical benthic microalgae. Mar. Pollut. Bull..

[51] Pesce S (2010). Evaluation of single and joint toxic effects of diuron and its main metabolites on natural phototrophic biofilms using a pollution-induced community tolerance (PICT) approach. Aquat. Toxicol..

[52] Grossmann K (2010). Auxin herbicides: current status of mechanism and mode of action. Pest Manage. Sci..

[53] Vinyard DJ, Ananyev GM, Charles Dismukes G, Photosystem II (2013). The reaction center of oxygenic photosynthesis. Annu. Rev. Biochem..

[54] Chesworth J, Donkin M, Brown M (2004). The interactive effects of the antifouling herbicides Irgarol 1051 and Diuron on the seagrass Zostera marina (L.). Aquat. Toxicol..

[55] Krieger-Liszkay A, Rutherford AW (1998). Influence of herbicide binding on the redox potential of the quinone acceptor in photosystem II: relevance to photodamage and phytotoxicity. Biochemistry.

[56] Krieger-Liszkay A (2005). Singlet oxygen production in photosynthesis. J. Exp. Bot..

[57] Osborn HL, Hook SE (2013). Using transcriptomic profiles in the diatom *Phaeodactylum tricornutum* to identify and prioritize stressors. Aquat. Toxicol..

[58] Mercurio P (2018). Contribution of transformation products towards the total herbicide toxicity to tropical marine organisms. Sci. Rep.

[59] Millie DF, Hersh CM, Dionigi CP (1992). Simazine-induced inhibition in photoacclimated populations of *Anabaena circinalis* (Cyanophyta). J. Phycol..

[60] Guasch H, Sabater S (1998). Light history influences the sensitivity to atrazine in periphytic algae. J. Phycol.

[61] Tang J, Hoagland KD, Siegfried BD (1998). Uptake and bioconcentration of atrazine by selected freshwater algae. Environ. Toxicol. Chem..

[62] Descolas-Gros C, Oriol L (1992). Variations in carboxylase activity in marine phytoplankton cultures. ß-carboxylation in carbon flux studies. Mar. Ecol. Prog. Ser.

[63] Ramakrishnan B, Megharaj M, Venkateswarlu K, Naidu R, Sethunathan N (2010). The impacts of environmental pollutants on microalgae and cyanobacteria. Crit. Rev. Environ. Sci. Technol..

[64] Ralph P, Smith R, Macinnis-Ng C, Seery C (2007). Use of fluorescence-based ecotoxicological bioassays in monitoring toxicants and pollution in aquatic systems. Toxicol. Environ. Chem..

[65] Büchel C, Wilhelm C (1993). *In vivo* analysis of slow chlorophyll fluorescence induction kinetics in algae: progress, problems and perspectives. Photochem. Photobiol..

[66] Schubert, N. *Phototprotective mechanisms in red algae*, Ph. D. thesis. Universidad Autónoma de Baja California, Mexico, (2008).

[67] MacColl, R. Phycobilisomes: a structure-function model. In: MacColl, R., 2018. Phycobiliproteins. 9–23 (2018).

[68] Apt KE, Collier JL, Grossman AR (1995). Evolution of the phycobiliproteins. J. Mol. Biol..

[69] Magnusson, M. *Effects of priority herbicides and their breakdown products on tropical, estuarine microalgae of the Great Barrier Reef Lagoon. Ph. D. thesis*, James Cook University, Australia, (2009).

[70] Huerlimann R, Heimann K (2013). Comprehensive guide to acetyl-carboxylases in algae. Crit. Rev. Biotechnol..

[71] Tu, M., Hurd, C. & Randall, J. M. Weed Control Methods Handbook, The Nature Conservancy, https://www.invasive.org/gist/products/handbook/methods-handbook.pdf (accessed 12 September 2019) (2001).

[72] Durkin, P. & Follansbee, M. Imazapic-human health and ecological risk assessment-final report. Prepared for USDA Forest Service, Forest Health Protection. (2004).

[73] Tang CY, Huang Z, Allen HC (2010). Interfacial water structure and effects of Mg2+ and Ca2+ binding to the COOH headgroup of a palmitic acid monolayer studied by sum frequency spectroscopy. J. Phys. Chem. B.

[74] Brzozowska A, Duits MH, Mugele F (2012). Stability of stearic acid monolayers on Artificial Sea Water. Colloids Surf. Physicochem. Eng. Aspects.

[75] Posthuma, L., Suter, G. W. II & Traas, T. P. Species sensitivity distributions in ecotoxicology. *CRC press* (2001).

[76] Negri AP (2019). Adjusting tropical marine water quality guideline values for elevated ocean temperatures. Environ. Sci. Technol..

[77] Hill DR, Wetherbee R (1989). A reappraisal of the genus Rhodomonas (Cryptophyceae). Phycologia.

[78] Fleeger JW, Carman KR, Nisbet RM (2003). Indirect effects of contaminants in aquatic ecosystems. Sci. Total Environ..

[79] Hammer A, Schumann R, Schubert H (2002). Light and temperature acclimation of *Rhodomonas salina* (Cryptophyceae): photosynthetic performance. Aquat. Microb. Ecol..

[80] Guevara M, Arredondo-Vega BO, Palacios Y, Saéz K, Gómez PI (2016). Comparison of growth and biochemical parameters of two strains of *Rhodomonas salina* (Cryptophyceae) cultivated under different combinations of irradiance, temperature, and nutrients. J. Appl. Phycol..

[81] Barlow SB, Kugrens P (2002). Cryptomonads from the Salton Sea, California. Hydrobiologia.

[82] Guillard RR, Ryther JH (1962). Studies of marine planktonic diatoms: I. *Cyclotella nana* Hustedt, and *Detonula confervacea* (Cleve) Gran. Can. J. Microbiol.

[83] Stone S, Adams M, Stauber J, Jolley DF, Warne MSJ (2019). Development and application of a multispecies toxicity test with tropical freshwater microalgae. Environ. Pollut..

[84] Trenfield MA (2015). Aluminium, gallium, and molybdenum toxicity to the tropical marine microalga Isochrysis galbana. Environ. Toxicol. Chem..

[85] Pease, C., Mooney, T., Trenfield, M., Costello, C. & Harford, A. Updated procedure for the 72 hour algal growth inhibition toxicity test using *Chlorella* sp. Internal Report 645, Department of the Environment and Energy, https://www.environment.gov.au/science/supervising-scientist/publications/internal-reports/updated-procedure-algal-growth-inhibition-toxicity-test (2016).

[86] Franklin, N., Stauber, J., Markich, S. & Lim, R. A new tropical algal test to assess the toxicity of metals in freshwaters. Supervising Scientists Report 133. (1998).

[87] OECD. Organisation for Economic Cooperation and Development (OECD) guidelines for the testing of chemicals: freshwater alga and cyanobacteria, growth inhibition test. Test No. 201, https://search.oecd.org/env/test-no-201-alga-growth-inhibition-test-9789264069923-en.htm (2011).

[88] Schreiber U, Müller JF, Haugg A, Gademann R (2002). New type of dual-channel PAM chlorophyll fluorometer for highly sensitive water toxicity biotests. Photosynth. Res.

[89] Mercurio P, Mueller JF, Eaglesham G, Flores F, Negri AP (2015). Herbicide persistence in seawater simulation experiments. PLoS One.

[90] Mercurio, P. III Herbicide persistence and toxicity in the tropical marine environment, University of Queensland, School of Medicine. 148 pp, 10.14264/uql.2016.722 (2016).

[91] Fisher, R., Ricardo, G. & Fox, D. jags NEC: A Bayesian No Effect Concentration (NEC) package, https://github.com/AIMS/NEC-estimation (accessed 03 december 2019) (2019).

[92] Fox DR (2010). A Bayesian approach for determining the no effect concentration and hazardous concentration in ecotoxicology. Ecotoxicol. Environ. Saf.

[93] Warne MSJ, King O, Smith RA (2018). Ecotoxicity thresholds for ametryn, diuron, hexazinone and simazine in fresh and marine waters. Environ. Sci. Pollut. Res..

[94] USEPA. ECOTOX User Guide: ECOTOXicology Database System. Version 5.0. United States Environmental Protection Agency, http://cfpub.epa.gov/ecotox/, (accessed 05 September 2019) (2019).

[95] Hook SE (2014). RNA-Seq analysis of the toxicant-induced transcriptome of the marine diatom, Ceratoneis closterium. Marine genomics.

[96] His, E. & Seaman, M. Effect of twelve pesticides on larvae of oysters (*Crassostrea gigas*) and on two species of unicellular marine algae (*Isochrysis galbana* and *Chaetoceros calcitrans*). *ICES, Copenhagen, Denmark* (1993).

[97] McFeters GA, Bond PJ, Olson SB, Tchan Y (1983). A comparison of microbial bioassays for the detection of aquatic toxicants. Water Res.

