# Supplementary material for: Toxicity of ten herbicides to the tropical marine microalgae Rhodomonas salina
Source: Sci Rep. 2020 May 6;10:7612. doi: 10.1038/s41598-020-64116-y (PMC7203118; doi:10.1038/s41598-020-64116-y)
Supplement: Supplementary file 1 — Supplementary information. [file 41598_2020_64116_MOESM1_ESM.pdf]

Toxicity of ten herbicides to the tropical marine microalgae *Rhodomonas salina*

Marie C. Thomas[1,2*], Florita Flores[1,2], Sarit Kaserzon[3], Rebecca Fisher[4] & Andrew P. Negri[1,2]

[1]Australian Institute of Marine Science, Townsville, QLD 4810, Australia
[2]AIMS@JCU: Australian Institute of Marine Science, College of Marine and Environmental Sciences, James Cook University, Townsville, Queensland 4811, Australia
[3]Queensland Alliance for Environmental Health Sciences (QAEHS), The University of Queensland, Woolloongabba, QLD 4102, Australia
[4]Australian Institute of Marine Science, Indian Ocean Marine Research Centre, University of Western Australia, Crawley, WA 6009, Australia

[*]m.thomas@aims.gov.au

Table S-1. Summary of the chemical properties of herbicides used in this study, stock solutions, and nominal and measured concentrations. A solvent carrier (SC) was used in the diuron (ethanol), simazine (ethanol), and haloxyfop (DMSO) toxicity test. The solvents were chosen based on their relative ability to dissolve the herbicides at the concentrations required. Seawater controls (SWC) were used for the remaining tests. Log Kow values from PubChem (https://pubchem.ncbi.nlm.nih.gov/). Solubilities in water (at 20 °C) are compiled using the Pesticide Properties Database (https://sitem.herts.ac.uk/aeru/ppdb/). Stock solutions were prepared in milli-Q water or filtered seawater (FSW). Measured concentrations are the average of initial and final concentration in each test. All concentrations in µg L^{-1}.

**Herbicide**	**Solubility (mg L^{-1})**	**log KOW**	**Stock solution (mg L^{-1})**	**FSW/milli-Q**	**Nominal concentration**		**Measured concentration**	
					Growth test	PAM test	Growth test	PAM test
Inhibition of photosynthesis at PSII								
Diuron	35.6	2.68	10	milli-Q	SWC; SC; 0.3; 1; 2; 4; 6; 10; 20	SWC; SC; 0.5; 1; 2; 4; 6; 10; 20	SWC; SC; 0.30; 0.98; 1.97; 3.93; 5.90; 9.83; 19.97	SWC; SC; 0.49; 0.98; 1.97; 3.93; 5.90; 9.83; 19.97
Metribuzin	1165	1.7	10	milli-Q	SWC; 0.3; 1; 3; 10; 30; 100	SWC; 0.3; 1; 3; 10; 30; 100	SWC; 0.27; 0.88; 2.65; 8.85; 26.55; 88.49	SWC; 0.27; 0.88; 2.65; 8.85; 26.55; 88.49
Hexazinone	33000	1.85	5	milli-Q	SWC; 1; 3; 6; 12; 25; 75	SWC; 1; 3; 6; 12; 25; 75	SWC; 0.86; 2.57; 5.15; 10.3; 21.5; 64.4	SWC; 0.86; 2.57; 5.15; 10.3; 21.5; 64.4
Bromacil	815	2.11	10	milli-Q	SWC; 0.3; 1; 3; 10; 30; 100; 300	SWC; 0.3; 1; 3; 10; 30; 100; 300	SWC; 0.26; 0.86; 2.57; 8.56; 25.7; 85.6; 257	SWC; 0.26; 0.86; 2.57; 8.56; 25.7; 85.6; 257
Tebuthiuron	2500	1.79	10	milli-Q	SWC; 1; 3; 10; 30; 100; 300	SWC; 1; 3; 10; 30; 100; 300	SWC; 0.98; 2.94; 9.80; 29.40: 98.00; 294	SWC; 0.98; 2.94; 9.80; 29.40: 98.00; 294
Simazine	5	2.18	10	FSW	SWC; SC; 1; 3; 10; 30; 100; 300; 600	SC; 1; 3; 10; 30; 100; 300; 600	SWC; SC; 0.62; 1.86; 6.21; 18.62; 62.07; 186.20; 372.41	SC; 0.62; 1.86; 6.21; 18.62; 62.07; 186.20; 372.41
Propazine	8.6	2.93	8	FSW	SWC; 1; 3; 10; 30; 60; 100; 200; 300	SWC; 1; 3; 10; 30; 60; 100; 200; 300	SWC; 0.65; 1.96; 6.53; 19.6; 39.2; 65.3; 131; 196	SWC; 0.65; 1.96; 6.53; 19.6; 39.2; 65.3; 131; 196

Inhibition of acetohydroxyacid synthase (AHAS)								
Imazapic	2230	0.393	2,000	FSW	SWC; 10,000; 30,000; 60,000; 100,000; 250,000; 500,000	SWC; 30,000; 60,000; 100,000; 250,000; 500,000	SWC; 15,935; 47,806; 95,611; 159,352; 398,380; 796,760	SWC; 47,806; 95,611; 159,352; 398,380; 796,760
Inhibition of acetyl-CoA carboxylase (ACCase)								
Haloxyfop	1.6	NA	100	FSW	SWC; SC; 2,000; 4,000; 6,000; 8,000; 10,000; 16,000	SC; 2,000; 4,000; 6,000; 8,000; 10,000; 16,000	SWC; SC; 464; 927; 1,391; 1,854; 2,318; 3,708	SC; 464; 927; 1,391; 1,854; 2,318; 3,708
Action like indole acetic acid (plant hormone) promoting uncontrolled growth								
2,4-D	23180	2.81	600	FSW	SWC; 1,000; 3,000; 10,000; 30,000; 100,00; 300,000; 600,000	SWC; 1,000; 3,000; 10,000; 30,000; 100,00; 300,000	SWC; 931; 2,793; 9,309; 27,928; 93,093; 279,279	SWC; 931; 2,793; 9,309; 27,928; 93,093

Table S-2. Slope and goodness of fit of concentration-response curves for specific growth rate (SGR) and effective quantum yield ($\Delta F/F_m'$) data from Fig. 1 Sigmoidal, 4 parameter model. NA indicates values could not be calculated.

**Herbicide**	**SGR**		**$\Delta F/F_m'$**	
	Slope	R^2	Slope	R^2
Diuron	1.9	0.99	1.6	0.99
Metribuzin	1.4	0.99	1.4	0.99
Hexazinone	2.9	0.98	1.9	0.99
Bromacil	1.6	0.99	1.2	0.99
Tebuthiuron	1.6	0.99	1.2	0.99
Simazine	1.4	0.99	1.2	0.99
Propazine	1.5	0.97	1.2	0.99
Imazapic	1.9	0.97	NA	NA
Haloxyfop	NA	NA	NA	NA
2,4-D	NA	NA	NA	NA
